# Observations on the Medical Treatment of Insanity

**Published:** 1833-01-01

**Authors:** 


### Observations on the Medical Treatment of Insanity. By *E. J. Seymour*, M.D. Physician to St. George's Hospital, &c.

This little volume contains the substance of Dr. Seymour's Croonian Lectures, delivered at the College in 1831, and the author considers himself much indebted to the learned treatise of Dr. Guislain, of Ghent, who has written with much talent on maniacal diseases.

Dr. S. bespeaks indulgence for his observations on insanity, on account of the extreme difficulty of the enquiry, and the darkness in which it is enveloped. A drawback on the progress of mental pathology is, he thinks, to be found in the circumstance of insanity being chiefly attended to by exclusive practitioners in that branch of the healing art—" medical men who resign the care of other diseases, and, with few and eminent exceptions, lose the power of investigating the aberrations of intellect, in conjunction with the other functional diseases of the human frame."

Dr. Seymour aims only at the merit of a faithful and judicious compiler—at laying before the public " an outline of the labours of others"—an analysis of what has been recommended with the view of simplifying our knowledge of causes, and determining upon what cases are, and what cases are not, remediable in the present state of the medical art. Although insanity is too often incurable, yet, under judicious treatment, a considerable proportion of patients recover. Thus, Mr. Warburton's establishment contains 400 lunatics. In the year 1829, of 200 admitted, 50 were discharged in the course of the year. Instances are given, from other authorities, of the cure of insanity under apparently desperate circumstances.

Dr. S. next adverts to the division of those cases which result from moral, and those from physical causes. The physical causes of insanity are very puzzling. We see great organic diseases of the brain without insanity—and great insanity, without any organic affection appreciable by the senses. Still we must suppose that functional disorder of the brain exists, and is essential to, the phenomena of mental derangement. In the brain, as in the stomach, liver, kidneys, and other organs, intense functional disturbance may exist, and that for a considerable time, without any change of structure that can be demonstrated on dissection. This is the key to the difficulty respecting the pathology of insanity.

Dr. S. dwells very properly on the disturbances of the cerebral functions, from sympathy with various other organs, as the stomach, heart, liver, &c.

" But the most evident causes for the disturbance of the functions of the brain, from sympathy with the disturbed functions of other parts, are found in the various changes which occur in the generative system. In women, from the commencement of puberty to the termination of that period, when the uterus becomes unfit for its specific purposes, the brain is liable to be disturbed by every change, whether healthy or diseased, of the uterine system. In early life, when the catamenia first occur, at each returning month, in delicate girls principally, the mind is affected. If imperfectly established, great bodily pain, with violent headaches, arise; if the catamenia are profuse at an early age, quick and irregular action of the heart and arteries, faintings, fretfulness, and visionary alarms, and even epileptic fits, ensue. Some females are obliged at this period to seclude themselves entirely from the family; their minds being disturbed in every gradation, from feelings of distress or discontent to absolute aberration of intellect. Who shall attempt to describe all the variations of spasmodic disease which attend this period of life? Sometimes the disturbance of the mind is shewn in the numerous forms of imposition attempted by the patient on the attending practitioner. Sometimes inordinate pains are complained of in the region of the bladder; and if the disease be doubted, the patient will have recourse to some means, in her own idea conclusive, to convince all beholders: thus persons, at this period of life, have professed to have passed gravel, or sand, which, on examination, proved that it never could have been generated or contained in an animal body. At other times, inordinate vomiting is the symptom for which medical advice is required; and it has occurred to me, as it has doubtless to others, to find this incessant vomiting kept up by substances taken for the very purpose by the patient herself. Sometimes the patient cannot swallow; at other times she loathes food, and will exist on almost incredibly small quantities of it: and yet these patients have received an education which would make them shun falsehood on any other subject, and are of a rank in life where nothing was to be gained by pity, except that commiseration, attention, and astonishment, which excite and occupy the mind.

Is it possible to conceive such cases, otherwise than the result of an alteration in the mental faculties nearly allied to mania? As far as I know, such cases always occur in young females, and mostly in persons labouring under some deviation from a healthy condition of the menstrual discharge: I have never met with, nor heard of a case of this description, in women who have borne children." 23.

Among the physical causes of insanity, the introduction into the system of alcohol, opium, and mercury, is generally mentioned by authors—the former more especially. Moral causes, however, are far more productive of insanity than physical causes. They are very numerous, including almost the whole of the passions, and the various mental emotions, dolorous or joyful, arising from the accidents and diversified scenes of life. Of 442 cases of mania, examined by Esquirol, 121 arose from physical, and 321 from moral causes. Other physicians, however, have not found the proportion of moral causes so great.

In the second chapter, Dr. S. discusses the moral causes, which are chiefly grief, jealousy, ambition, terror, and superstition. Monomania, mania, and amentia are defined, and examples of each adduced. The most imperfect of the external senses —hearing, is that which is most frequently affected. Nothing is more common than to find men asserting that they have communications with the invisible world —that spirits whisper to him—or that animals abuse him—or that his enemies employ tubes, constructed on acoustic principles, to goad him into madness. Dr. Seymour saw a lady who heard, with her left ear, swearing and obscene expressions.

The memory is often affected, and may be either increased, diminished, or destroyed. In monomania it is generally increased; in mania, it varies from great strength to great feebleness. Patients restored to reason often remember, with remarkable exactness, what befel them while under restraint.

It is well known that monomania is the most difficult form of insanity to discover, the patient often concealing his infirmity or delusion, especially if he imagines that the inquirer is endeavouring to detect it. Instances of this kind are innumerable, and have even been portrayed by novelists and dramatists. We may refer to Mackenzie's Man of Feeling for an instance. Dr. S. is led from this consideration to another—the expediency, or otherwise, of confining a monomaniac. It would appear that such cases frequently end in suicide. Dr. S. mentions the case of a lady, who had contracted suspicions, that those about her harboured malignant intentions towards her. Suddenly, and in the night, she arose, and threw herself from her window into the area, breaking both her legs by the fall. Dr. S. insists on the propriety of great caution in examining monomaniacs. They may display the greatest powers of mind, till, by chance, the cracked cord is struck. Then is the melody marred, and the discordance becomes apparent. Dr. S. expresses, then, no decided opinion on confinement or non-confinement. It would obviously be mere twaddle to lay down a dogmatic rule. An intelligent man will be guided by the circumstances of the particular case, in spite of any dogma, and a fool will not be the better for a rule, which must necessarily be half eaten up with exceptions.

Is it better to remove patients from their homes and usual habits? As a general thing it most undoubtedly is so; the common experience of men has decided it. But, under peculiar combinations of circumstances, an opposite plan may be adopted. The remarks of the late Dr. Gooch on this subject, and the case of the lady, told in his characteristic style, must be fresh in the minds of many of our readers. The cases in which this is recommended by Dr. G. are those in which the patient is not recovering, but when month after month elapses without amendment, and the mental delusions assume a shape accessible to moral impressions. We must not expect much from this method.

Dr. S. next adverts to coercion. There has no doubt been a good deal of cant on this subject; yet still it is a distinguishing and a gratifying feature of the state of society, that their feelings or their prejudices, say which you will, have abolished the employment of violence and cruelty, and rendered the corporeal management of the insane both more humane and more philosophical. The balance of good inclines greatly to this side, though now and then a maniac may abuse the gentleness of those who have the charge of him. Dr. Heberden's predilection for cribs and straw may, for aught we know, be very just; but certain we are, that it is better for mankind that the feelings both of medical men and the public should run counter to those agreeable receptacles for madmen. Dr. Seymour feels convinced, from observation, that polished iron handcuffs are more merciful engines of coercion than leathern gloves; or, in the cases of the very violent, waistcoats. Dr. Seymour reprobates, feelingly and eloquently, the abominable maxim of the Augustan Celsus—"Ubi perperàm aliquid dixit aut fecit, fame, vinculis, plagis coercendus est."

Of all the remedies applicable to derangement from moral causes, employment and exercise are perhaps the best. All are agreed on this point. Bodily exercise seems, for evident reasons, most applicable to monomania. In mania it is chiefly useful during the lucid interval and convalescence; and even during the paroxysm, if it can be strongly exercised until some degree of weariness can be induced, calm, tranquillity, and sleep, not unfrequently ensue. The kindlier feelings and affections of the mind, when excited, tend to banish melancholy. In the upper classes, especially among females, the tending of domestic animals, rabbits, pigeons, &c. has been found serviceable, and Dr. S. found many of these means resorted to in the Quaker's Retreat, near York.

Dr. S. next alludes to the power of enduring cold which maniacs are said to possess. We think it probable that this is greatly exaggerated. It is well known that man under the influence of strong emotions, or under the physical influence of stimulants, as alcohol, will resist to a certain degree, the operation of external irritants or injuries. Maniacs, in some cases, are nearly similarly circumstanced, their minds exclusively directed to some object, or occupied with one feeling, or their whole frame under the influence of maniacal passion or excitement. In such it is likely that cold will be comparatively innocuous, or that blisters or cauteries may be applied unregarded. But we doubt whether this immunity be general, and we are happy to perceive that this view is taken by Dr. Seymour.

"Notwithstanding such observations and examples, gangrene not unfrequently seizes the extremities when frostbitten, and the usual diseases of diarrhœa, and inflammatory affections of the thoracic viscera, not unfrequently attack maniacal patients. The best explanation of these apparently contradictory facts is to be found in the perverted mind of the patient; his limbs suffer, for their sensibility is probably not really altered from the natural condition. But the disordered mind perceives not the bodily ailment, or, to use the illustration of a foreign writer, a blister applied does not, perhaps, attract a momentary attention from the maniac, but it does not the less produce inflammation and suppuration." 56.

It has often been a matter of surprise, that an invasion of mania occasionally disperses or mitigates other maladies, and, inversely, that an attack of acute disease will suspend or altogether remove mania. That this is occasionally the fact there can be no question, nor need it occasion our astonishment. Is it not known that pregnancy will at times suspend the march of phthisis, that acute diseases will put a stop to pregnancy, and cause abortion? Do we not see cold produce inflammation of the lungs, and check gout, and the external appearance of gout relieve internal inflammation? Is it not on the principle implied in the frequency of these facts, that we found the employment of our long list of counter-irritants? In short, there is nothing surprising in the matter. Epidemic diseases are also said to have seldom prevailed in lunatic asylums. But this may be owing to their seclusion, their regularity of habits and of diet, rather than to any preservative power on the part of madness. The cholera has prevailed in several lunatic establishments. Dr. Seymour relates several curious and interesting cases of mania relieved or removed by other diseases. We need not dwell any longer on the subject.

The third chapter or lecture is occupied with the consideration of treatment, or the application of medicine to the cure of insanity.

We must first, of course, investigate the causes, and ascertain, as far as possible, whether there be or be not organic affection of the brain. If there are very evident symptoms of vascular excitement, antiphlogistic measures, and blood-letting may be had recourse to, but with caution. The experience of all who have had the management of the insane on a great scale is against blood-letting. Pinel speaks strongly against it. In what are even called the *high* cases, it is not the vascular system that is powerfully excited—it is the nervous system. Bleed largely, nay, in some cases bleed at all, and you produce idiotcy, or typhoid collapse. Let us hear what say Messrs. Beverley and Phillips, the gentlemen who have the charge of the patients in Mr. Warburton's establishment.

"The number of patients admitted with vascular excitement, requiring blood-letting, are very few indeed; we seldom or ever use the lancet in cases of excitement, if there is no evident effect upon the brain from increased arterial action, so as to lead us to fear an approaching attack of apoplexy or paralysis. The reason we do not use the lancet in cases without any such symptoms existing of disease going on in the brain, is, that we have done so in several instances, and the result was not favourable; the patient became reduced from the loss of blood, and the excitement not abated; the powers of the constitution gave way, the tongue became typhoid, and the patient sank into a state of collapse, and died." 69.

Bleeding then is in the gross inapplicable to these cases. What are we to do? There is a great mass of experience in favour of cold, which may be used in the form of ice—of the shower-bath—or of the douche. Dr. S. relates two instances of the beneficial effects of ice applied to the head He found it very useful in the low maniacal delirium attending the epidemic typhus that prevailed some little time ago amongst the poor of London. The following is the report of Messrs. Beverley and Phillips on the shower-bath.

"We have found, in some cases, the shower-bath of great service; but it appears to us, from the experience we have had of it, that it is more beneficial in cases of a very violent nature, with increased vascular excitement, as we have given it a trial in cases of various descriptions, and in some without the slightest benefit.

There was a case admitted into this Establishment, evidently a case that was completely cured by the use of it. A gentleman, aged 30, small, and of a light complexion, who had been studying hard, and constantly confined to one room, was attacked with furious mania; thought he had found out perpetual motion, and that he could make the sun stand still. Pulse very quick, 120, and small; pupils contracted; imagined he could reach any thing he saw, and grasped at them; incessantly talking; tongue furred and dry. We ordered him to go into the shower-bath. His extreme violence put us so on our guard, that we got six keepers to take him there. We persuaded him, with much difficulty, to go in, by saying it was only a sentry-box. On hearing that, he immediately went; the door was closed and secured; the shock was so unexpected, that he screamed, and held his breath for a short time after the shock was over; then gasped, and knocked the sides and the door into pieces, and stepped out; but was immediately secured, rubbed dry, and put to bed. He had a little refreshing sleep during the night. In the morning he vowed vengeance against the doctors for murdering him. We could not prevail upon him this day to go into the bath, which obliged us to confine and carry him there. He bore the shock better; was taken out, rubbed, and put to bed. Slept better; the tongue appeared cleaner, and he was not so violent. Bowels open; he begged to be released from confinement, which was complied with. He took a little exercise; we put him in the shower-bath almost without any difficulty; sleep returned; gave him a dose of calomel and colocynth; more rational; he inquired what had been the matter; thought he had been asleep, and in the evening begged himself to go into the bath and have more medicine. From that time he became tranquil, took mild aperients, and was discharged well in a fortnight from the date of his admission.

We have had several cases, nearly of the same nature, where the shower-bath in its results proved invaluable." 73.

Thus it appears that the shower-bath is chiefly applicable to cases of dementia. Dr. S. has not found the douche much employed in our establishments.

As cold is useful in mania, so the warm-bath appears to have been beneficial in melancholia. Several testimonies are given in favour of it. Blistering or an eruption produced by tartar-emetic on the scalp have been found useful. At Mr. Warburton's establishment these means have been abandoned, in consequence of their tendency to induce erysipelas. In robust habits an eruption from tartar-emetic over the biceps humeri has proved more advantageous. Tartar-emetic, as an emetic, has done good in melancholia, in doses sufficient to keep down the circulation, it has been serviceable in paroxysmal mania.

Our author passes to the consideration of opium, "the greatest blessing ever accorded to mankind." We think there have been greater, but let them pass. Dr. Seymour labours ingeniously to explain the various opinions on its powers. Somehow or other the world are not contented with it, and chemist after chemist has laboured to fix its good principle and separate its bad. Hence the many preparations of opium, hence the forms of morphia. Let us look at the experience of Messrs. Phillips and Beverley on this head. It is an interesting extract.

" 'We have found the acetate of morphia useful both in the excited and the low form of insanity. We have also found it useful in cases of fixed delusions, but not of any great standing, and more useful in the low than the excited form of the disease. Of five cases of melancholy, three got well; the remaining two are certainly improving under the use of this medicine. Of five cases of excitement, two were discharged cured; one remains much improved; two received no benefit. It is necessary to observe that we have used this medicine in several cases without taking notes, and the result was similar to the two cases mentioned, that is, without benefit. It appeared to us that morphia did not produce the same good effect in excited as in other cases, unless there was an occasional interval of reason. In the cases mentioned we have commenced with a fourth, and have not found it necessary to exceed half a grain. At present we have a patient taking half a grain dose every night with decided advantage, and we think the case very interesting, and proving the extraordinary effect of this medicine in cases of melancholy. A woman, of the age of 36, the mother of four children, was attacked with depression of spirits while pregnant of her last child. She did not feel the attack before she quickened, but immediately after she had a strong desire to destroy herself and children. This continued during pregnancy. After she was delivered she became worse, and attempted to commit suicide several times, and described her feelings, which is not common in such cases. She continued in this state, not fit to be trusted without a strict watch. She was sent here about two years ago; and what is extraordinary in her case is, that about noon all the feelings of desire of self-destruction left her. This occurred within the last three months, from which time they have remained the whole of the day. Various means were tried without effect. Our first idea, from the regularity of the attack, was to treat her as an intermittent, which failed. About a fortnight ago we gave her the morphia, beginning with a fourth of a grain, and gradually increasing it to half a grain: after taking the second dose, one-third of a grain, she slept all night; in the morning was cheerful, without feeling the propensity to destroy herself. The third day she had a return, which lasted until noon; the dose was then increased to half a grain. The fourth morning she had not any return, and continued well until the fifth day after the half-grain dose was given, when she had a return from five o'clock in the morning until nine, a paroxysm three hours shorter than any of the preceding. She is now free from any desire of destroying herself.'

The following is a case of the excited kind, in which this remedy was employed with advantage:—

'A. R. æt. 36, was admitted in February 1831, in a very high state of nervous excitement; she was a widow, and mother of four children. When admitted she was much excited, and constantly talking. Tongue dry; pulse very quick; skin moist. She was excited to such a degree that she tore the jacket and clothes to ribbons; refused her food; and would swallow nothing without force. She was ordered a pint of porter daily, with beef-tea and arrow-root. This diet was considered necessary, because if so much excitement continued without support she would fall into a state of collapse, and die. All our efforts were unavailing in giving her food. We determined to try the morphia. The first night it had not the least effect; she was noisy, screaming until morning; on the following day refused her food, and the excitement was unabated; we got the porter and arrow-root swallowed with some difficulty; the morphia was increased to half a grain; did not make any noise during the night, and appeared to be drowsy in the morning; but when she was spoken to answered in a very incoherent way, and the excitement continued; the porter and beef-tea was given with less trouble: the medicine repeated; slept well during the night; appeared, on questioning her in the morning, to have a slight return of reason, such as to inquire where she was; took her food better; tongue moist; pulse not so quick, and bowels open. Ordered two pints of porter, beef-tea, and arrow-root, as usual. Medicine repeated at night; slept very well; more rational; began to cry; took her food much better; drank the porter, and appeared to relish it: the medicine was repeated every night until the 6th of March, when she appeared perfectly well; the morphia was discontinued; she employed herself, and was discharged 14th April cured.*"

Our space is so limited that we must make short work of the remaining ten pages. In them are discussed the merits or demerits of hyosciamus, belladonna, hydrocyanic acid, arsenic, diffusible stimulants, especially camphor, purgatives, with an eulogy on the oil of croton, the oil of turpentine, and diet. Dr. Seymour agrees with those who have found an antiplogistic diet productive of evil. Full diet, even stimulants, are often productive of advantage in "high cases." Dr. Seymour winds up by recommending educated physicians to study insanity, and rescue an investigation so interesting and so honourable from the hands of quacks. He also tells them that they must often come between the public and their prejudices, and that, being thus awkwardly placed between two fires, they must not desert their colours. What those colours are it would be hard to say, but we fear they are not the *union-jack*.

In conclusion we need scarcely recommend this little volume to our readers. It does no discredit to Dr. Seymour's ingenuity, intelligence, and judgment. It is not so original as his work on the ovaria, which we had the pleasure of reviewing, but it is calculated to lead physicians to the study of insanity, and, what is better, to induce them to regard it as a curable rather than incurable disease. Both these aims are worthy of a philosophic and cultivated mind.

---

* "On inquiry, I find that the good effects of morphia still continue in cases in the White House. The muriate of morphia is now preferred, and is said to produce less nausea than the acetate.—Dose, gr. ¼.

